# Potential association between bacterial infections and ischemic stroke based on fifty case-control studies: A systematic review and meta-analysis

**DOI:** 10.1016/j.nmni.2022.100980

**Published:** 2022-04-15

**Authors:** M. Keikha, M. Karbalaei

**Affiliations:** 1) Antimicrobial Resistance Research Center, Mashhad University of Medical Sciences, Mashhad, Iran; 2) Department of Microbiology and Virology, Faculty of Medicine, Mashhad University of Medical Sciences, Mashhad, Iran; 3) Department of Microbiology and Virology, School of Medicine, Jiroft University of Medical Sciences, Jiroft, Iran

**Keywords:** Bacterial infection, *Chlamydia pneumonia*, *Helicobacter pylori*, ischemic stroke, meta-analysis, *Mycobacterium tuberculosis*, *Mycoplasma pneumonia*

## Abstract

**Background:**

Stroke is considered as one of the most important concerns in health care centers around the world. By definition there are two types of stroke including ischemic stroke and hemorrhagic stroke. Approximately three-quarters of stroke cases are ischemic strokes, which occur due to several risk factors such as hypertension, obesity, atherosclerosis, diabetes mellitus, osteoarthritis, and inflammatory responses. In recent years, infectious diseases have noticed as a new risk factor for ischemic stroke. Given the importance of the issue, some bacteria that cause chronic infections, especially *Chlamydia pneumonia*, *Helicobacter pylori*, *Mycoplasma pneumonia*, *Mycobacterium tuberculosis*, and *Coxiella burnetii* have been considered.

**Methods:**

In the present meta-analysis, we reviewed 50 case-control studies and assessed the possible association of bacterial infections with the occurrence of ischemic stroke.

**Results:**

We analyzed the information of 33,978 participants in several nested case-control studies, and ultimately showed that bacterial infections could increase the risk of ischemic stroke. Our results suggest that bacterial infections significantly increase in the risk of ischemic stroke (OR: 1.704; 1.57-1.84 with 95% CIs; *p* value = 0.01).

**Conclusions:**

In this meta-analysis, a significant relationship was observed between infection by three bacteria such as *C. pneumoniae*, *H. pylori*, and *M. tuberculosis* with the occurrence of ischemic stroke. Furthermore, due to the similarity between TLRVYK domain in β2-glycoprotein-I and TLRVYK peptide in various of microorganisms, produced antibodies against pathogens interact with β2-glycoprotein-I, hence the cross-reaction phenomenon increases the positive relationship between infectious diseases and ischemic stroke.

## Background

Nowadays stroke is accounted as one of the most striking complications of cardiovascular disorders, and is classified into two types, ischemic stroke and hemorrhagic stroke. The incidence of ischemic strokes is higher than hemorrhagic strokes, so that about 71% of strokes are ischemic and the rest are hemorrhagic [1]. In general, strokes are the second most common cause of death (approximately 13.7 million cases in 2016) worldwide [2]. This disease has become a global health concern, so it is estimated that one in four people will have a stroke during his/her lifetime [3]. Several risk factors such as obesity, hypertension, smoking, dyslipidemia, diabetes mellitus, alcohol consumption, atrial fibrillation, carotid stenosis, inflammation, and epigenetic events play a major role in the incidence of stroke [4].

Recently, the role of inflammatory reaction in the formation of vascular disorders such as atherosclerotic plaques, carotid intima-media thickness (CIMT), arterial wall disruption, and vascular wall instability has been well established [5]. Although the role of infectious agents as a risk factor for stroke is not well understood, but there is some evidence linking infection with microorganisms to disorders such as atherosclerotic lesions, metabolism imbalance, cardiovascular disease (CVD), and hypertension; the most probable pathogens include cytomegalovirus (CMV), hepatitis C virus (HCV), human immunodeficiency virus (HIV), herpes simplex virus type 1/2 (HSV 1/2), Epstein Barr virus (EBV), influenza virus, periodontal microflora, *Helicobacter pylori* (*H. pylori*), *Chlamydia pneumoniae* (*C. pneumoniae*), *Haemophilus influenza* (*H. influenza*), *Mycoplasma pneumoniae* (*M. pneumoniae*), *Mycobacterium tuberculosis* (*Mtb*), *Streptococcus pneumonia* (*S. pneumoniae*), *Coxiella burnetii* (*Coxiella burnetii*), Tannerella *forsythia* (*T. forsythia*) [[6], [7], [8], [9], [10], [11], [12], [13]]. Among all infectious agents, pathogenic bacteria play an important role in the development of CVD through their virulence mechanisms such as toxins, enzymes, interference in host immune response, as well as infective endocarditis [3,6]. According to the literature, endocarditis and sepsis are two main underlying diseases that increase the risk of stroke in humans [14].

The present meta-analysis was performed for the purpose of plausible relationship between ischemic stroke and infection with *H. pylori*, *C. pneumoniae*, *M. pneumoniae*, and *Mtb*. As well as, the potential role for stroke induction was estimated for each group of infectious agents.

## Methods

### Literature search strategy

Comprehensive systematic search was done through databases such as Scopus, PubMed, Cochrane Library, Embase, and Google scholar up to May 2020. For searching, we used keyword phrases such as “*Helicobacter pylori*” and “ischemic stroke,” “*Chlamydia pneumonia*” and “ischemic stroke,” “*Mycoplasma pneumonia*” and “ischemic stroke,” “*Mycobacterium tuberculosis*” AND “ischemic stroke,” as well as “bacterial infection” and “Ischemic stroke.” All published English articles were retrieved without limitation on the date of publication. The search strategy was performed by two authors separately; in case of disagreement, the third author judged and decided.

### Study selection criteria

**Fig. 1:**
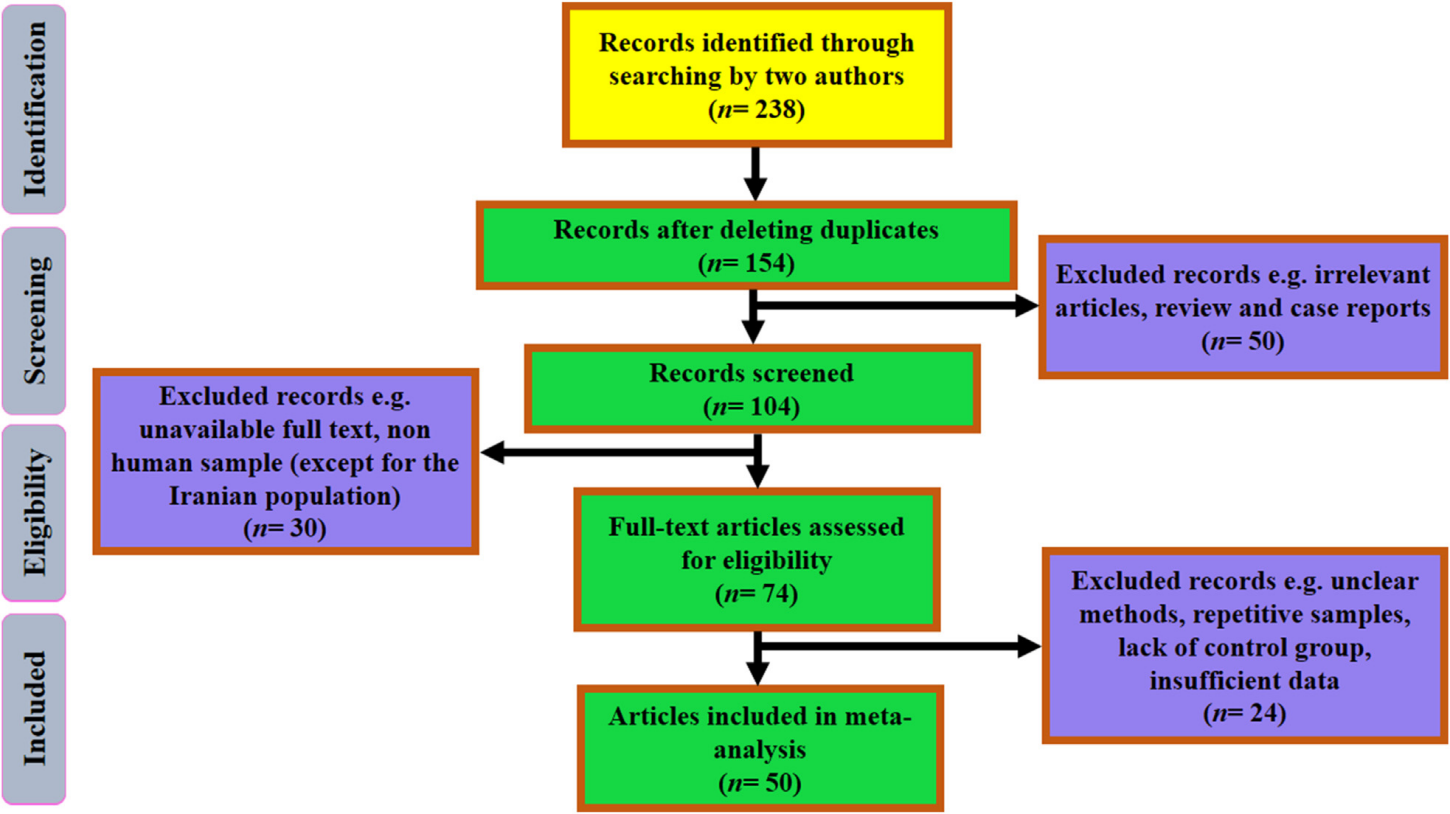
The flowchart of included studies.

In the present meta-analysis inclusion criteria included: 1) case-control studies on the role of bacterial infections in ischemic stroke; 2) studies on the role of infection by *H. pylori*, *C. pneumoniae*, *M. pneumoniae*, and *Mtb* in ischemic stroke; 3) studies with standard diagnostic methods including ELISA and other immunoassays, conventional microbiology methods, PCR, blotting assay, and urease breath test (UBT); 4) clarity in the results of included studies. In addition, our exclusion criteria included case-report, letter to editor, review, congress abstract, non-English texts, prospective or cohort, post-stroke infections studies, repetitive results, and unclear studies. The flowchart of included studies is presented in Fig. 1.

### Quality assessment and data collection

The quality assessment of included studies was evaluated using the Newcastle–Ottawa scale (NOS). Required information included first author, publication year, location of each studies, type of infection agents, diagnostic methods of infection, age, gender, case group, control group, and number of infected cases in both case and control groups (Table 1) [[15], [16], [17], [18], [19], [20], [21], [22], [23], [24], [25], [26], [27], [28], [29], [30], [31], [32], [33], [34], [35], [36], [37], [38], [39], [40], [41], [42], [43], [44], [45], [46], [47], [48], [49], [50], [51], [52], [53], [54], [55], [56], [57], [58], [59], [60], [61], [62], [63], [64]].

**Table 1:** Characteristics of included studies

First author	Year	Area	Age (in years)		Gender (F/M)		Case group (n)	Control group (n)	Microorganism type	Number of bacterial infections (n)		Diagnostic method	Ref
			case	control	case	control				case	control		
Wincup	1996	UK	54	53.5	NA	NA	137	136	*H. pylori*	93	78	ELISA	[15]
Cook	1998	UK	67.9	56.5	73/103	674/844	176	1518	*C. pneumoniae*	81	280	MIF	[60]
Rasura	2000	Italy	NA	NA	NA	NA	101	101	*C. pneumoniae*	26	8	ELISA	[65]
Elkind	2000	USA	68.5	68.5	47/42	47/42	89	89	*C. pneumoniae*	72	74	ELISA	[16]
Grau	2001	Germany	62	59.5	36/73	27/55	109	82	*H. pylori*	57	34	ELISA	[17]
Heuschmann	2001	Germany	74.6	74.6	77/68	138/122	145	260	*H. pylori*	67	117	ELISA	[61]
Ponzetto	2002	Italy	56.7	57.4	22/58	88/232	80	320	*H. pylori*	64	190	UBT	[18]
Pietroiusti	2002	Italy	63.2	63.9	11/50	89/62	61	151	*H. pylori*	43	106	PCR	[19]
Majka	2002	Germany	NA	NA	NA	NA	80	80	*H. pylori*	69	54	ELISA	[20]
Madre	2002	Spain	70	70	46/45	53/59	91	112	*C. pneumoniae*	40	34	IFI	[21]
Tarnacka	2002	Poland	74	66	91/88	66/56	179	122	*C. pneumoniae*	6.00 (95% CI: 1.61-22.29)		ELISA	[22]
Tanne	2003	Israel	61	61	8/126	8/126	134	134	*C. pneumoniae*	115	110	ELISA	[23]
Muller	2003	Denmark	69	45	84/109	125/243	193	368	*C. pneumoniae*	20	29	PCR	[24]
Kawamoto	2003	Japan	75	74	17/23	48/37	40	85	*C. pneumoniae*	29	52	ELISA	[25]
Moayyedi	2003	UK	70.5	70.2	228/239	227/161	467	388	*H. pylori*	274	206	ELISA	[27]
Voorend	2004	Netherlands	43.9	39.4	22/19	31/24	41	55	*C. pneumoniae*	28	38	ELISA	[29]
Ngeh	2004	UK	80	80	59/41	57/25	100	82	*M. pneumoniae*	95	82	ELISA	[30]
Gabrielli	2004	Italy	68	66	56/49	66/64	105	130	*H. pylori*	75	81	ELISA	[26]
Anzini	2004	Italy	34.6	36.5	60/81	90/102	141	192	*C. pneumoniae*	2.9 (95% CI: 1.77-4.76)		ELISA	[31]
Ngeh	2005	UK	NA	NA	59/41	60/27	100	87	*C. pneumoniae*	71	57	ELISA	[28]
Masoud	2005	Iran	64.3	61.7	43/48	40/40	91	80	*H. pylori*	59	36	ELISA	[32]
Kongoji	2005	Japan	63.5	62.7	7/6	2/5	13	7	*C. pneumoniae*	5	0	PCR	[33]
Wohlschlaeger	2005	Germany	65.1	73.3	5/5	16/7	7	21	*C. pneumoniae*	4	1	PCR	[34]
Sawayama	2005	Japan	71.5	69	22/40	95/48	62	143	*H. pylori*	9.68 (95% CI: 3.56–33.08)		UBT	[35]
Johnsen	2005	Denmark	60.4	60.5	99/155	99/155	254	254	*C. pneumoniae*	1.28 (95% CI: 0.83–1.95)		ELISA	[36]
Elkind	2006	USA	72.3	72.3	125/121	219/38	246	474	*C. pneumoniae*	156	257	ELISA	[37]
Park	2006	Korea	66.7	66.8	62/63	62/63	125	125	*H. pylori*	100	75	ELISA	[38]
Njamnshi	2006	Cameroon	NA	NA	64/0	64/0	64	64	*C. pneumoniae*	41	35	ELISA	[39]
Jozwiak	2007	Poland	44	40	40/54	44/59	94	103	*C. pneumoniae*	63	15	ELISA	[40]
Ashtari	2008	Iran	65.4	60.2	43/38	24/19	81	43	*H. pylori*	57	29	ELISA	[41]
Lin	2008	Taiwan	64.2	63.2	202/248	198/252	450	450	*C. pneumoniae*	334	257	ELISA	[42]
Bandaru	2008	India	47.8	47.8	149/51	149/51	200	200	*C. pneumoniae*	72	35	ELISA	[43]
Bastiani	2008	Italy	76.6	76.5	51/55	51/55	106	106	*H. pylori*	67	57	UBT	[44]
Bandaru	2009	India	35.3	35.3	30/90	30/90	120	120	*C. pneumoniae*	35	15	ELISA	[45]
Gagliardi	2009	Brazil	NA	NA	25/40	37/22	65	59	*C. pneumoniae*	0	1	PCR	[46]
Sheu	2010	Taiwan	33.5	42.4	824/1459	2939/3910	2283	6849	*M. tuberculosis*	136	256	Culture	[57]
Mousavi	2011	Iran	65.6	62.9	46/50	36/57	96	93	*H. pylori*	44	39	ELISA	[47]
Rai	2011	India	53.6	38.6	16/35	14/34	51	48	*C. pneumoniae*	32	38	ELISA	[48]
Ķēniņa	2011	Latvia	65.8	64.3	41/61	22/26	102	48	*C. pneumoniae*	64	17	ELISA	[49]
Hasan	2011	Iraq	58.02	56.1	18/32	18/22	50	40	*C. pneumoniae*	36	21	ELISA	[50]
Bandaru	2012	India	74	71	30/70	32/68	100	100	*C. pneumoniae*	29	16	ELISA	[51]
Hassanein	2014	Egypt	53	52.6	35/55	25/35	90	60	*H. pylori*	70	32	ELISA	[66]
Eini	2014	Iran	68.9	66.9	60/81	60/81	141	141	*C. pneumoniae*	111	74	ELISA	[54]
Ebrahimi-Rad	2014	Iran	NA	NA	NA	NA	27	25	*C. pneumoniae*	20	13	ELISA	[55]
Wu	2014	Taiwan	53	53.2	1922/3882	1925/3879	5804	5804	*M. tuberculosis*	176	207	Culture	[56]
Srivastava	2014	India	43.6	43.2	NA	NA	80	80	*C. pneumoniae*	42	26	ELISA	[58]
Sagar	2016	India	47.8	46.6	14/25	8/22	39	30	*H. pylori*	26	12	ELISA	[59]
Roham	2016	Iran	69.1	67.2	61/36	51/46	97	97	*M. pneumoniae*	4	0	ELISA	[62]
Salmasi	2017	Iran	66.7	65.9	38/32	39/31	70	70	*H. pylori*	61	51	ELISA	[63]
Mrđen	2017	Croatia	72.8	72.8	34/32	34/32	82	93	*H. pylori*	21	32	ELISA	[64]

### Quantitative synthesis

Data analysis was performed using Comprehensive Meta-Analysis (CMA) software version 2.2 (Biostat, Englewood, NJ). For this purpose, first the frequency of each bacterial infection including *H. pylori*, *C. pneumoniae*, *M. pneumoniae*, and *Mtb* was measured and according to the event rate (%) was reported for both case and control groups. Next, using the Odds ratio (OR) with 95% Confidence intervals (CIs), potential role of bacterial infection in the occurrence of ischemic stroke was analyzed. Moreover, by Cochran's *Q* and *I*^*2*^ statistic parameters, we analyzed heterogeneity of included studies. By our default, the cases with Cochrane *Q* statistic p < 0.1 and *I*^*2*^ > 25% were described as cases with high heterogeneity. According to the Dersimonian and Laird method, the random effect model and the fixed effect model were applied in high and low heterogeneity cases, respectively. Finally, Egger's regression was used for estimating asymmetry of funnel plot and also publication bias.

## Results

### Characterization of included studies

**Fig. 2:**
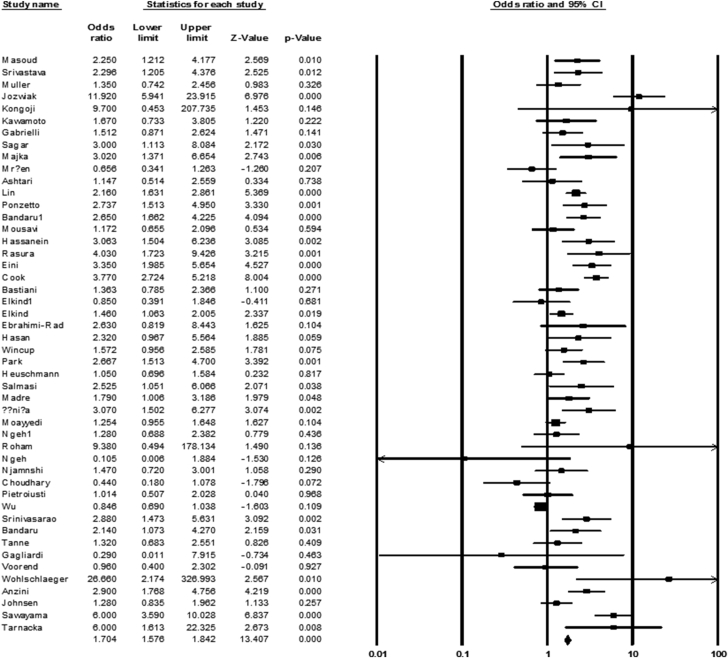
The forest plot of the meta-analysis on the association between bacterial infection and ischemic stroke.

After the initial search, 238 documents were identified from 1996–2017, and finally, 50 studies were selected based on inclusion criteria. In a large number of eligible studies, the presence of bacterial infection at the time of acute ischemic stroke was investigated; however, there were also cohort studies that evaluated the longitudinal effects of bacterial infections on ischemic stroke. Of these studies, 28, 18, 2, and 2 studies were related to *C. pneumoniae*, *H. pylori*, *M. pneumoniae*, and *Mtb*, respectively. In addition, the diagnostic methods were included ELISA, PCR, UBT, MIF, IFI, and conventional microbiology. In the present study, the information of 33,978 individuals including 13,652 patients (case) and 20,326 healthy (control) was reviewed. Average of age in case and control groups was 61.7 and 59.8, respectively. The frequency of men in both case and control groups was measured 62.6% and 56.1%, respectively. According to statistical analysis, the presence of bacterial infection in both case (ischemic stroke) and control groups was 38% (37-39 with 95% CIs) and 26% (25-27 with 95% CIs), respectively. We also found a meaningful relationship between bacterial infections and the incidence of ischemic stroke (OR: 1.704; 1.57-1.84 with 95% CIs; *p* value = 0.01; *I*^*2*^ = 78.55; Q-value: 219.11; df = 47; Egger's intercept = 1.23) (Fig. 2).

### The potential association between C. pneumonia infection and ischemic stroke

**Fig. 3:**
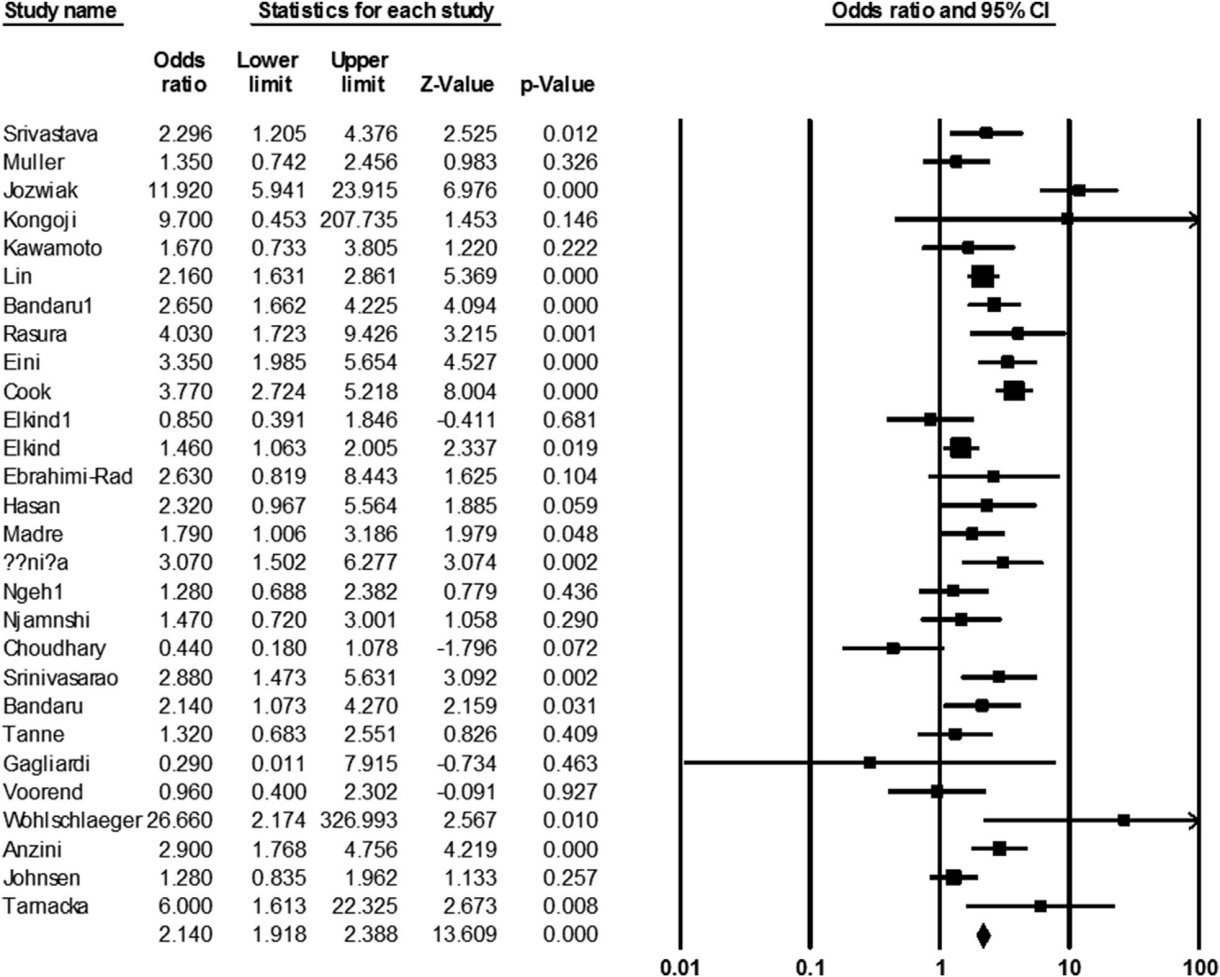
The forest plot of the meta-analysis on the association between *C. pneumoniae* infection and ischemic stroke.

We found 28 articles about the role of infection by *C. pneumonia* in the incidence of ischemic stroke. The rate of infection was estimated at 57% (54–59 with 95% CIs) and 36% (34–37 with 95% CIs) in both stroke and healthy groups, respectively. A significant relevance was observed between infection by *C. pneumonia* and ischemic stroke (OR: 2.14; 1.91–2.38 with 95% CIs; *p*-value = 0.001; *I*^2^ = 71; Q-value = 93.29; df = 27; Egger's intercept = 0.06) (Fig. 3).

### The potential association between H. pylori infection and ischemic stroke

**Fig. 4:**
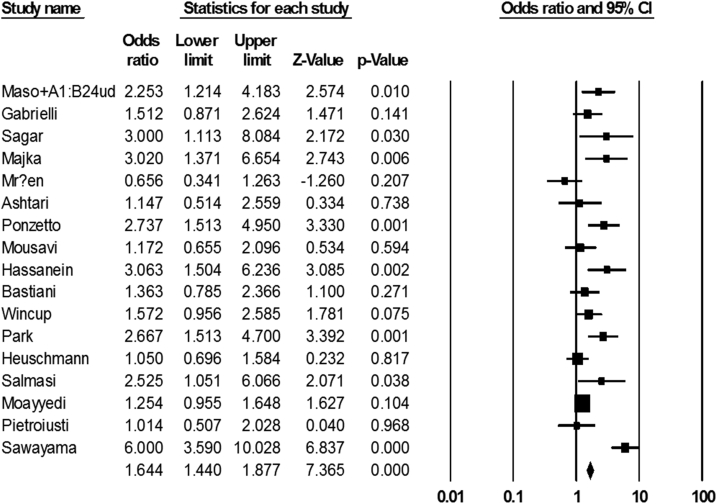
The forest plot of the meta-analysis on the association between *H. pylori* infection and ischemic stroke.

Of the 50 case-control articles included in this meta-analysis, 18 were conducted on the association between *H. pylori* infection and the incidence of ischemic stroke. The infection rate in both patient and healthy groups was 63% (60–65 with 95% CIs) and 55% (52–57 with 95% CIs), respectively. In accordance with statistical results, it seems that there is a meaningful relationship between infection by *H. pylori* and ischemic stroke (OR: 1.64; 1.44–1.87 with 95% CIs; *p*-value = 0.001; *I*^*2*^ = 72.88; Q-value = 59; df = 16; Egger's intercept = 1.87) (Fig. 4).

### The potential association between M. pneumonia infection and ischemic stroke

**Fig. 5:**
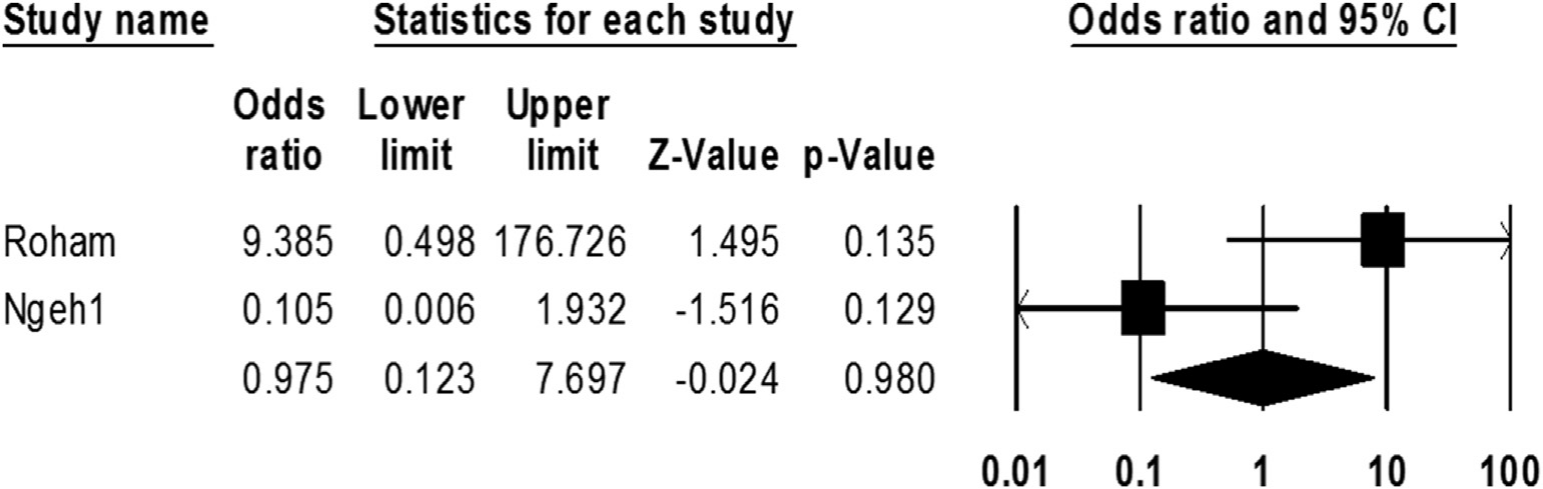
The forest plot of the meta-analysis on the association between *M. pneumoniae* infection and ischemic stroke.

Regarding the plausible role of infection by *M. pneumonia* and occurrence of ischemic stroke, we retrieved only two eligible studies. The incidence rate of infection in both case and control groups was 55% (39–70 with 95% CIs) and 47% (11–86 with 95% CIs), respectively. We did not find any significant relationship between *M. pneumonia* infection with ischemic stroke (OR: 0.97; 0.12–7.69 with 95% CIs; *p*-value = 0.98; I2 = 77.94; Q-value = 4.53; df = 1) (Fig. 5).

### The potential association between M. tuberculosis infection and ischemic stroke

**Fig. 6:**
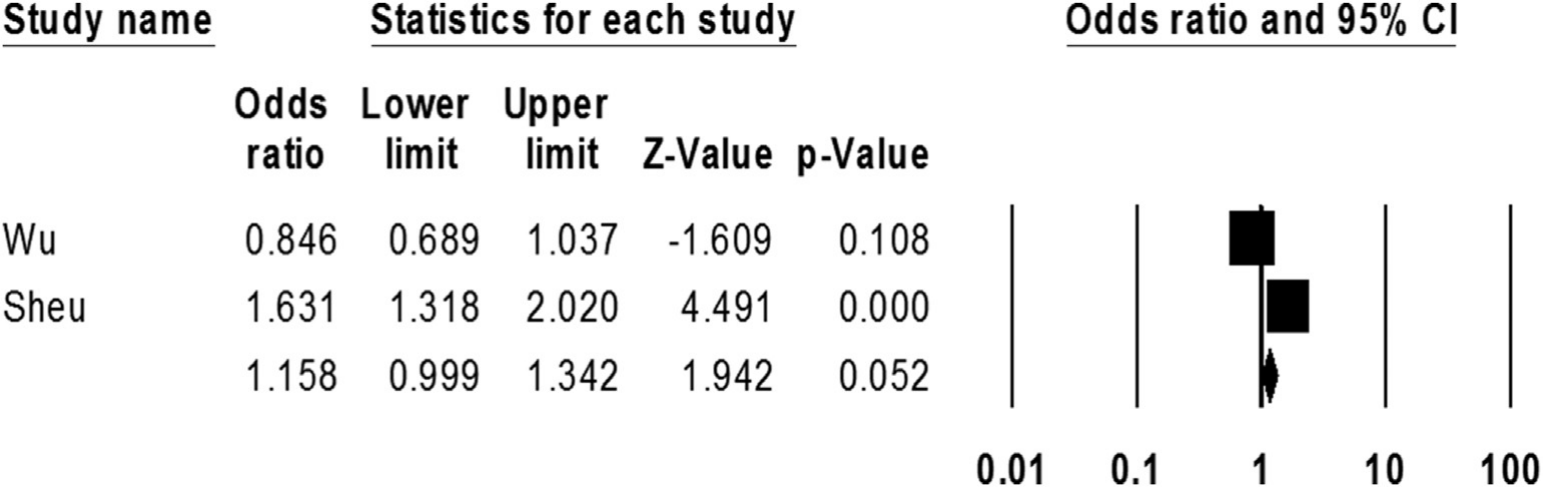
The forest plot of the meta-analysis on the association between *M. tuberculosis* infection and ischemic stroke.

Finally, to evaluate the association between infection by *Mtb* and stroke, we analyzed two studies. Based on statistical analysis, the rate of infection by *Mtb* in both ischemic and healthy groups was 4% (3.6–4.5 with 95% CIs) and 3% (3.3–4 with 95% CIs), respectively. However, we observed a significant relationship between infection by *Mtb* and the incidence of ischemic stroke (OR: 1.15; 0.99–1.34 with 95% CIs; *p*-value = 0.05; I2 = 94.73; Q-value = 18.98; df = 1) (Fig. 6).

In general, in the present study we appraised the potential role of infections by four bacteria including *C. pneumoniae*, *H. pylori*, *M. pneumoniae*, *Mtb*, and progression to ischemic stroke. In this meta-analysis we demonstrated a meaningful relationship between infection by three bacteria *C. pneumoniae*, *H. pylori*, and *Mtb* with occurrence of ischemic stroke. However, significant heterogeneity was observed between the studies, which in turn led to the unreliability of the current results; moreover, there were differences in items such as bacterial identification methods (i.e. instrument or materials), study design, as well as difference in time-point contributed between included studies. Unfortunately, we did not access to raw data to provide subgroup analysis to reduce heterogeneity. Therefore, more extensive research is needed to validate the current analysis.

## Discussion

**Fig. 7:**
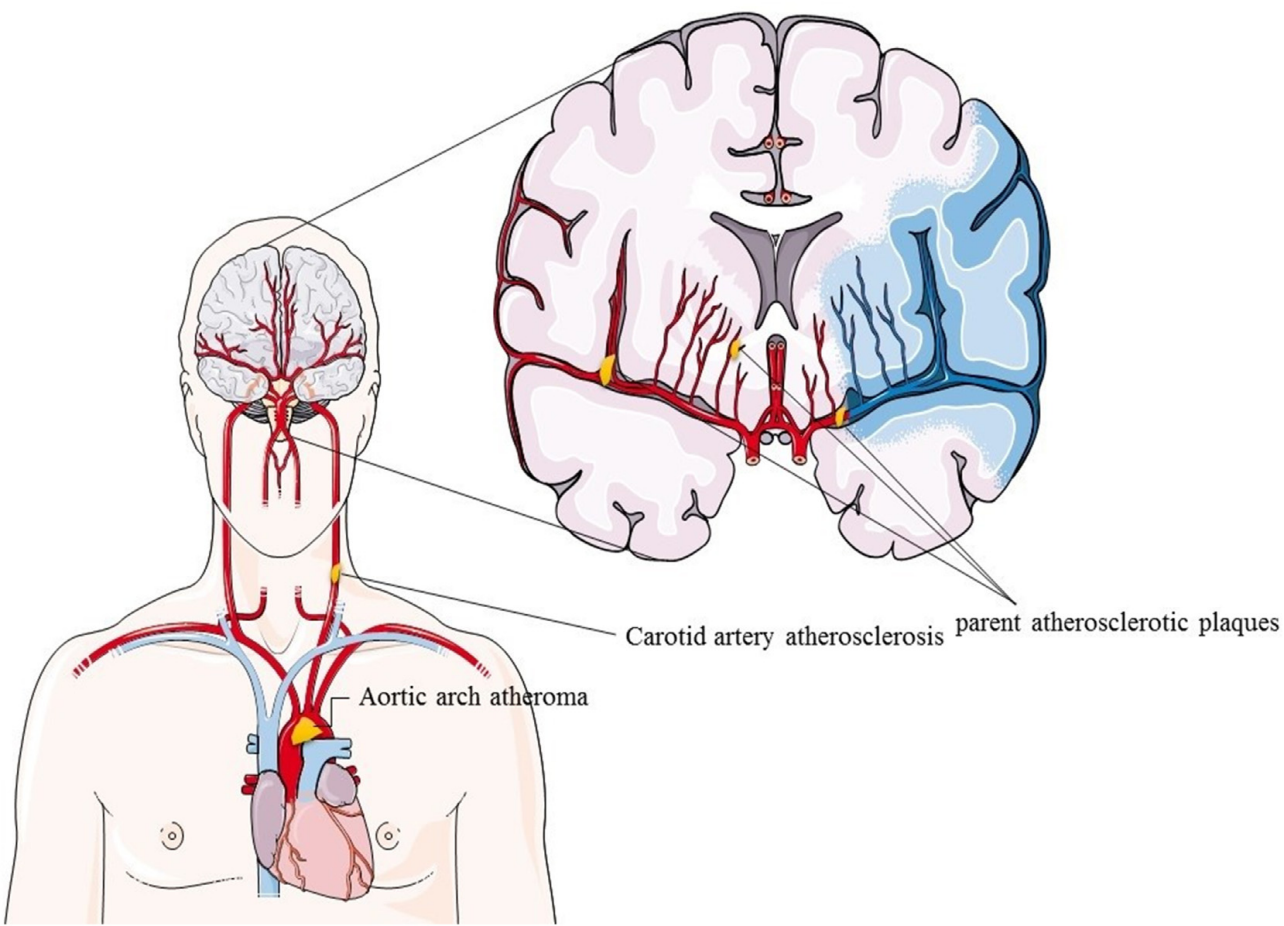
Cardiovascular disorders caused by infection. Bacterial infections cause some cardiovascular disorders including parent atherosclerosis plaques, Carotid artery atherosclerosis, and Aortic arch atheroma. This figure was taken from the website https://smart.servier.com/image-set-download/.

Stroke is one of the most common cardiovascular disorders, and its occurrence depends on underlying risk factors including hypertension, diabetes mellitus, smoking, hyperlipidemia, atrial fibrillation, atherosclerosis, as well as characteristics such as age, gender, and family history [67]. Depending on the circumstances, the risk factors for the onset of stroke are different, so the mechanism of the increase in the incidence of stroke in young people and its trend towards autumn and winter is not yet fully understood [68]. In recent decades, understanding the role of acute and chronic infections in stroke has received more attention; obviously, infection can lead to inflammation, which in turn causes complications such as the formation of fatty plaques in blood vessels, atherogenic reactions, and changes in host metabolism (Fig. 7) [69].

According to the literature, the infections caused by pathogens such as *C. pneumonia, H. pylori*, *M. pneumonia*, *Coxiella burnetii*, HIV, HSV 1-2, and CMV are more significant, these infectious microorganisms are often isolated from atherosclerotic plaques [12,70]. On the other hand, induction of CIMT and a high seropositive status in the population with CVD confirm this phenomenon [71]. Today, there is ample evidence of the role of bacterial infections in increasing stroke; for example, infectious endocarditis can lead to embolism and arthritis. Moreover, bacterial meningitis and chronic brucellosis cause vasculitis and thrombosis in cerebral arteries; likewise, rickettsial infections lead to damage of small vascular endothelial cells and eventually ischemic stroke [68].

Induced autoantibodies during infectious diseases are considered pathogenic autoantibodies and potentially interact with the phospholipid binding protein β2-glycoprotein-I (β2GPI), the major autoantigen in APS [72]. Due to the phenomenon of “molecular mimicry,” microbes mimic the natural proteins of the host with the help of their chemical structures [73]. In their study, Blank et al. showed that there is a high homology between the TLRVYK hexapeptide in β2GPI and various bacteria (*Pseudomonas aeruginosa*, *Yersinia pseudotuberculosis*, and *Streptomyces lividans*), viruses (CMV, polyoma virus, and adeno virus-40), yeasts (*Saccharomyces cerevisiae*), and parasites (*Schistosoma mansoni*), which in turn induces pathogenic H-3 anti-β2GPI mAb against β2GPI [74,75]. In the present study using statistical analysis of fifty case-control studies, we found that there is a significant relationship between bacterial infection and ischemic stroke cases (OR: 1.7; CI: 1.5–1.8).

*C. pneumonia* is a Gram-negative intracellular bacterium that was first introduced in 1980s. More than half of the world's population is infected with this bacterium; this fact has been confirmed by serological evidence [76,77]. In many studies, researchers have isolated *C. pneumonia* from carotid plaques, atherosclerotic plaques, and circulating leukocytes [77]. Sander et al. showed that eliminating *C. pneumoniae* infection could prevent the progression of CIMT [78]. Clinical trial studies have also suggested that treatment of this infection can potentially reduce vascular lesions in patients [79]. In our study the rate of infection by *C. pneumonia* in both case and control groups was measured 57% and 36% respectively. Also, we showed that there is a meaningful relationship between infection by this bacterium and occurrence of ischemic stroke (OR: 2.14; CI: 1.9–2.3).

*H. pylori* is a Gram-negative, helical, and microaerophilic bacterium that colonizes almost half of the world's population in the gastric mucosa [80]. The rate of infection by this bacterium is higher in developing countries than in developed countries, accordingly, in some parts of Africa the infection with this pathogen reaches 100% [81,82]. This bacterium is considered as a causative agent in disorders such as chronic gastritis, gastric ulcer, as well as gastric cancer; extra-gastrointestinal diseases related to this pathogen have attracted much attention, in which the association between *H. pylori* infection and CVD is well known [83,84]. In addition to isolating these bacteria from atherosclerotic plaques, their infection causes disorders such as insulin resistance, dyslipidemia, hypertension, and alteration in metabolism, all of which describe the potential role of *H. pylori* infection in increasing ischemic stroke, particularly nonembolic ischemic stroke [85,86]. Statistically, infection with this pathogen was measured at 63% and 55% in ischemic stroke patients and healthy individuals, respectively (OR: 1.6; CI: 1.4–1.8).

*M. pneumonia* is a respiratory pathogen that despite a poor understanding of its pathogenicity, many people have anti-*M.pneumonia* antibodies (IgG and IgM). Recent studies have shown its role in extra-pulmonary manifestations such as musculoskeletal, gastrointestinal, neurological, dermatologic, hematologic, and cardiovascular complications [87,88]. According to the literature, about 0.1% of patients infected with *M. pneumonia* develop neurological disability during their lifetime [89]. Vasculopathic lesions following this bacterial infection indicate the role of *M. pneumonia* in increasing the risk of ischemic stroke [68,90]. Two studies in this meta-analysis were related to the role of *M. pneumonia* in ischemic stroke susceptibility; infection by this bacterium was 55% and 47% in both case and control groups, respectively. Also, based on statistical analysis, no significant association was found between infection by *M. pneumonia* and ischemic stroke (OR: 0.97; CI: 0.12–7.6). However, the small number of studies may affect the present results, as only two studies had been performed on this bacterium. In addition, high heterogeneity causes instability of the results and we need more studies to confirm these findings.

*Mtb* is a life-threatening pathogen, and although this bacterium is commonly identified as a major cause of pulmonary tuberculosis, it also causes extra-pulmonary manifestations [91,92]. In recent years the role of bacterium in promoting neurological manifestation has been demonstrated, so that following arterial invasion, malignant vasculitis can be occurred during the tubercular meningitis [68]. Salindri et al. conducted a cohort study to show the effects of *Mtb* infection on chronic non-communicable disease; they found that previous tuberculosis (TB) infection could significantly increase susceptibility to ischemic stroke [93]. Our results also confirmed the possible association between TB infection and an increased risk of ischemic stroke.

*Coxiella burnetii* is an intracellular and Gram-negative coccobacillus bacterium which causes Q-fever, a zoonosis disease; Q-fever is endemic worldwide, especially in European countries e.g. Spain, France, and Germany [94]. Based on the findings of Vinacci et al., *Coxiella burnetii* is one of the main bacterial isolates in patients with infective endocarditis (IE) and acute ischemic stroke (AIS) [95]. During primary infection, *Coxiella burnetii* induces high levels of antiphospholipid antibodies, especially IgG anticardiolipin antibodies (IgG aCL), which in turn lead to antiphospholipid syndrome (APS); infectious IgG aCL are associated with several complications such as fever, thrombocytopenia, valvular heart disease, as well as chronic endocarditis [[96], [97], [98]]. In addition, serological markers indicate that there is a significant relationship between *Coxiella burnetii* infection and CVD in the elderly people [99,100].

Nevertheless, our study had several limitations which we must mention: 1) the small number of included studies; 2) lack of access to raw data to perform modulatory analysis to describe significant heterogeneity; 3) the effects of mixed infections are likely to be underestimated in qualified studies; 4) asymmetry of funnel plot also suggests the presence of significant publication bias within the included studies; 5) there was also difference in diagnostic method, study design, studies time-point, population ethnicity and location of included studies that actively contributed in heterogeneity of the included studies. In our study, a large number of eligible studies were cross-sectional that assessed the bacterial infections at the time of ischemic stroke, while, cohort studies could better deliberate the clear association between bacterial infection and susceptibility to ischemic stroke. Therefore, to confirm the results of the present study, we need more comprehensive studies.

## Conclusion

In the present study, the information of two nested case-control studies about the role of *Mtb* in ischemic stroke was evaluated. The rate of infection by this pathogen in both cases and control groups was 4% and 3%, respectively, and we found a significant association between mycobacterial infection and ischemic stroke (OR: 1.1; CI: 0.99–1.34). We assessed the relationship between bacterial infections and the development of ischemic stroke. Nevertheless, due to limitation in results, we could not evaluate the role of pre-existing risk factors in our research. Overall, for understanding the role of bacterial infections in ischemic stroke, it is better to perform a comprehensive study about the association between bacterial infections and traditional ischemic stroke risk factors, CIMT, atherosclerosis, and cardiovascular risk factors such as LDL and HDL. Our results indicate the need for additional longitudinal investigations to determine the impact of infectious disease on the risk of ischemic stroke; such studies will require substantial follow-up time and control subjects. We recommend further larger cohort studies to determine bacterial infections and their determining role in ischemic stroke. As we noted above, TLRVYK domain in β2GPI is the main peptide in cardiolipins structures and is homologous with TLRVYK peptide in various bacteria, viruses, yeasts, and parasites. Pathogenic autoantibodies (H-3 anti-β2GPI mAb) produced against infectious microorganisms cross-react with this peptide in β2GPI, thus may lead to ischemic stroke.

## Ethics approval and consent to participate

Not applicable (this article was provided based on researching in global databases).

## Consent to publish

All authors have informed consent about the content of this article.

## Availability of data and materials

All data will be available for anyone who requests those.

## Transparency declaration

There is no any conflict of interest among the all authors.

We have not received any funding for this research.

## Authors' contributions

1. MK1 was a major contributor in writing the manuscript.

2. MK2 was research director and translated this manuscript to English.

All authors read and approved the final manuscript.

## Abbreviations

CIMT: Carotid intima-media thickness
CVD: Cardiovascular disease
CMV: Cytomegalovirus
HCV: Hepatitis C virus
HIV: Human immunodeficiency virus
HSV 1-2: Herpes simplex virus type 1-2
EBV: Epstein Barr virus
NOS: Newcastle-Ottawa Scale
CMA: Comprehensive Meta-Analysis
OR: Odds ratio
CIs: Confidence intervals
*H. pylori*: *Helicobacter pylori*
*Mtb*: *Mycobacterium tuberculosis*
*C. burnetii*: *Coxiella burnetii*
*C. pneumoniae*: *Chlamydia pneumoniae*
*M. pneumoniae*: *Mycoplasma pneumonia*
